# Impaired neural structure and function contributing to autonomic symptoms in congenital central hypoventilation syndrome

**DOI:** 10.3389/fnins.2015.00415

**Published:** 2015-10-30

**Authors:** Ronald M. Harper, Rajesh Kumar, Paul M. Macey, Rebecca K. Harper, Jennifer A. Ogren

**Affiliations:** ^1^Brain Research Institute, University of California, Los AngelesLos Angeles, CA, USA; ^2^Department of Neurobiology, David Geffen School of Medicine, University of California, Los AngelesLos Angeles, CA, USA; ^3^Department of Anesthesiology, David Geffen School of Medicine, University of California, Los AngelesLos Angeles, CA, USA; ^4^Department of Radiological Sciences, David Geffen School of Medicine, University of California, Los AngelesLos Angeles, CA, USA; ^5^Department of Bioengineering, University of California, Los AngelesLos Angeles, CA, USA; ^6^UCLA School of Nursing, University of California, Los AngelesLos Angeles, CA, USA

**Keywords:** sympathetic, sudden death, depression, anxiety, blood pressure, magnetic resonance imaging, neuroprotection

## Abstract

Congenital central hypoventilation syndrome (CCHS) patients show major autonomic alterations in addition to their better-known breathing deficiencies. The processes underlying CCHS, mutations in the *PHOX2B* gene, target autonomic neuronal development, with frame shift extent contributing to symptom severity. Many autonomic characteristics, such as impaired pupillary constriction and poor temperature regulation, reflect parasympathetic alterations, and can include disturbed alimentary processes, with malabsorption and intestinal motility dyscontrol. The sympathetic nervous system changes can exert life-threatening outcomes, with dysregulation of sympathetic outflow leading to high blood pressure, time-altered and dampened heart rate and breathing responses to challenges, cardiac arrhythmia, profuse sweating, and poor fluid regulation. The central mechanisms contributing to failed autonomic processes are readily apparent from structural and functional magnetic resonance imaging studies, which reveal substantial cortical thinning, tissue injury, and disrupted functional responses in hypothalamic, hippocampal, posterior thalamic, and basal ganglia sites and their descending projections, as well as insular, cingulate, and medial frontal cortices, which influence subcortical autonomic structures. Midbrain structures are also compromised, including the raphe system and its projections to cerebellar and medullary sites, the locus coeruleus, and medullary reflex integrating sites, including the dorsal and ventrolateral medullary nuclei. The damage to rostral autonomic sites overlaps metabolic, affective and cognitive regulatory regions, leading to hormonal disruption, anxiety, depression, behavioral control, and sudden death concerns. The injuries suggest that interventions for mitigating hypoxic exposure and nutrient loss may provide cellular protection, in the same fashion as interventions in other conditions with similar malabsorption, fluid turnover, or hypoxic exposure.

## Introduction

The principal clinical and scientific interest in congenital central hypoventilation syndrome (CCHS) focuses on somatic ventilatory dysfunction, particularly the marked depression of respiratory musculature during sleep. However, the condition, as originally described by Mellins et al. (1970), is also accompanied by a range of autonomic symptoms (O'Brien et al., 2005). Those autonomic deficits vary in severity, lead to significant quality-of-life concerns, are frequently life-threatening, and almost certainly exacerbate the processes contributing to progressive neural injury in the syndrome. Moreover, the close integration of impaired autonomic processes with breathing also worsens the major feature in the syndrome, hypoventilation. Those impaired autonomic characteristics include dyscontrol of blood pressure, temperature, glucose, and CO_2_ sensing, and lead to reflexive influences on central respiratory control that interfere with normal breathing patterns. The hypoventilation, in turn, contributes to inadequate autonomic responses to challenges, a consequence of the close reflexive interactions between the somatomotor respiratory system and the sympathetic and parasympathetic systems. More insidiously, the autonomic and breathing dysfunctions, as well as central impairments native to the condition, lead to affective and central processing issues that must be addressed.

The objective of this examination of the autonomic nervous system in CCHS is to reveal the neural mechanisms underlying the major characteristics of the syndrome, to examine the impaired reflexive interactions contributing to those symptoms, and to suggest potential interventions which may be useful in managing the progression of the condition.

Determination of the genetic processes underlying the syndrome reveals that mutations of the paired-like homeobox (*PHOX2B*) gene, which contributes to early development of autonomic neurons, are principally responsible for the broad range of symptoms encountered (Amiel et al., 2003; Sasaki et al., 2003; Matera et al., 2004; Patwari et al., 2010; Weese-Mayer et al., 2010). Variations in expression of those mutations result in differing degrees of severity or uniqueness in characteristics found in the syndrome. Asymmetric pupillary innervation and high, poorly-regulated sympathetic tone are common, with little influence on heart rate variation from parasympathetic influences, as shown by high fixed heart rates (Woo et al., 1992) and syncope to straining efforts, e.g., toilet efforts. Profuse sweating and poor fluid control are also prevalent, as is impaired temperature regulation, with lower body temperatures, and an inability to maintain body temperature comfortably even on modestly warm summer nights (Vanderlaan et al., 2004). Disrupted autonomic control of the pupils is frequently accompanied by ancillary eye movement coordination issues. Some variations in expression of CCHS symptoms extend to loss of ganglia to the alimentary canal (Hirschsprung's disease; Bajaj et al., 2005), or long sinus pauses, requiring cardiac pacemaker intervention, and reduced control by parasympathetic efferents to the esophagus.

The primary concern for CCHS patients, however, is the loss of drive to the breathing musculature in the upper airway, thoracic wall, and diaphragm in the absence of other lung or muscle disease. The loss of central breathing drive is particularly enhanced during quiet sleep, while rapid eye movement sleep offers some degree of protection. Although the capability to exert voluntary breathing efforts during wakefulness is maintained, waking ventilation at rest can be a concern, with inactivity frequently accompanied by hypoventilation. The perception of high CO_2_ or low O_2_, and the sense of breathlessness, or dyspnea, is lost in CCHS patients. Losing this affective drive to breathe poses serious consequences; unless continually encouraged to breathe voluntarily, a waking, resting child may be at risk for hypoxia. Thus, overseers must note any signs of pallor when a child watches television or plays video games, and urge the child to voluntarily breathe (voluntary breathing remains intact). The brain structures mediating the perception of dyspnea, i.e., insula, cerebellum, cingulate (Banzett et al., 2000; Peiffer et al., 2001; Evans et al., 2002), are injured in CCHS patients (Kumar et al., 2005, 2012). The loss of the perception of breathlessness removes a major drive to breathe in the condition, and determining the mechanisms of the loss of that drive should provide insights into maintaining the integrity of breathing. That determination should also assist the opposite condition found in heart failure (HF) and chronic obstructive pulmonary disease, the enhanced perception of breathlessness that leads to a failure to exercise in those conditions.

A principal influence on breathing normally stems from thermal drive, as found in higher breathing rates or panting in some species. CCHS patients show low body temperatures (Vanderlaan et al., 2004), which contributes to a reduced drive to breathe. The loss of thermal drive in CCHS places affected patients at risk on very warm days, or if the child incurs a fever, sometimes requiring ancillary ventilatory support during those conditions.

One objective of this discussion of autonomic issues in CCHS is to show how the autonomic dysfunction emerges from central injury in the syndrome, and to demonstrate how the injury develops from *PHOX2B* mutations, as well as from hypoxic exposure, and more insidiously, from secondary consequences of the primary developmental injury to autonomic neurons. Some of the injuries in CCHS are progressive, likely an aftermath of hypoxia and these secondary consequences, and interventions may be available to prevent at least a portion of the damage.

## Central injury in CCHS underlying autonomic regulation dysfunction

**Figure 1:**
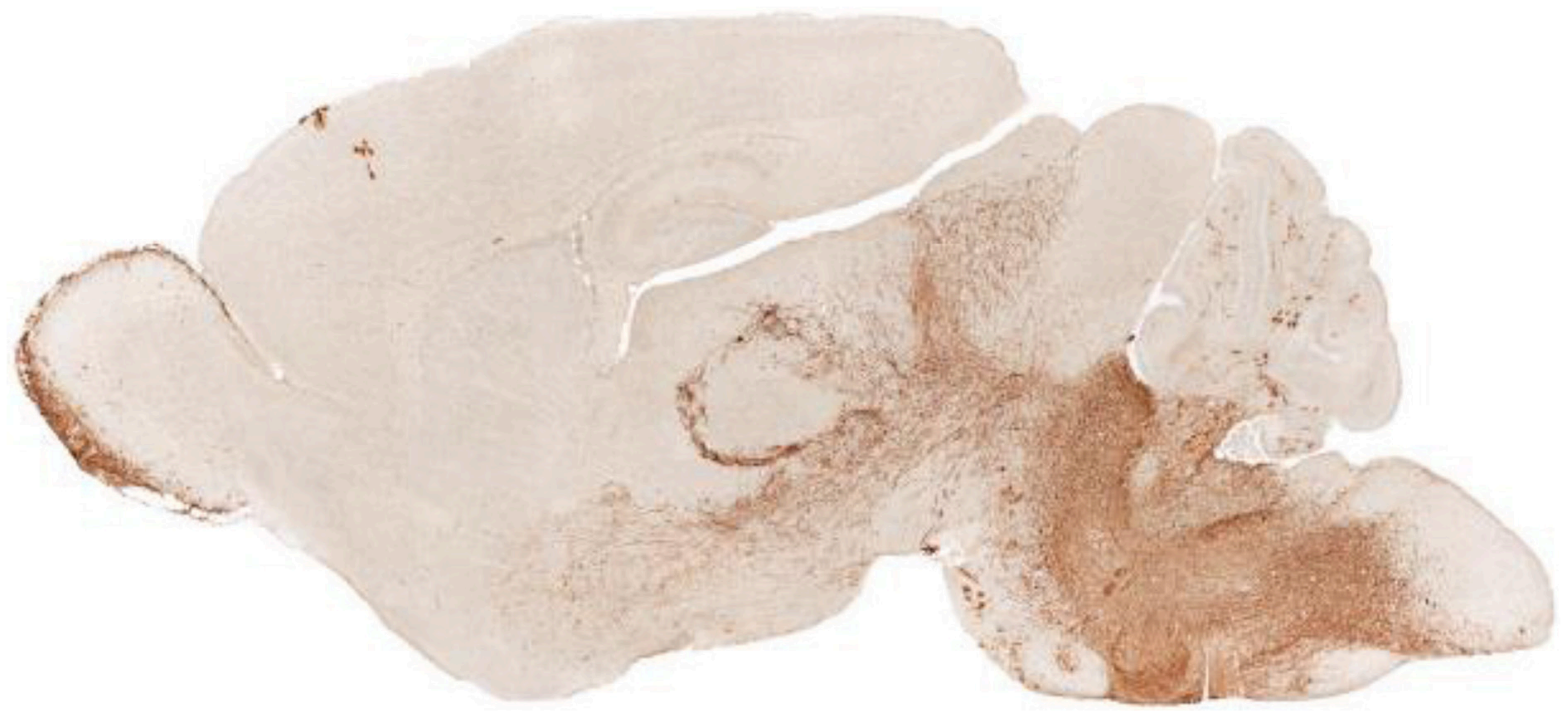
**Sagittal view of ***Phox2b*** distribution in a P7 ***Phox2b***-EGFP mouse (GENSAT)**. The distribution of expression sites appear in diencephalic brain areas, including the olfactory bulb, hypothalamus, and glia associated with axons; in the caudal brain the dorsal motor nucleus of the vagus, locus coeruleus, nucleus of the solitary tract, and area postrema are labeled.

The initial consequences of *PHOX2B* mutations significantly interfere with development of autonomic neurons prenatally. Maps of the distribution of *phox2b* in the embryonic mouse or P6 rat show a principal distribution in medullary and midbrain areas, but with a large distribution in cerebellar regions, and a remarkable distribution in glia of diencephalic regions, as demonstrated from the GENSAT^1^ images from Rockefeller University (Figure 1).

We assessed alterations in structure and function of the brain in CCHS patients in a series of studies using magnetic resonance imaging (MRI) techniques. The CCHS cases for these studies were diagnosed on the basis of clinical criteria used at the time of recruitment (American Thoracic Society, 1999), and thus, separation by extent of *PHOX2B* mutation was precluded. However, all of the subjects exhibited classic autonomic and breathing patterns characteristic of the condition, including ventilatory insensitivity to CO_2_ and nocturnal hypoventilation. These subjects ranged in age from 7 to 29 years, were principally under 19 years of age, and were compared to age- and gender-matched healthy controls. The studies revealed significant injury in a large number of brain sites; the structural injury was paralleled by impaired functional MRI (fMRI) responses to a series of ventilatory and autonomic challenges.

## Structural and functional injury to the ANS in adolescent CCHS children

The initial injury from *PHOX2B* mutations may have been sufficient to trigger the early hypoventilation, which even on short-term exposure, would elicit hypoxic injury sufficient to result in the damage found as early as the 7th year of life, the earliest age at which structural MRI evaluation could be performed in these sedation-free, waking studies. However, evaluation of injury several years apart on a limited number of subjects revealed progression of damage (Kumar et al., 2012). Thus, although the influences of *PHOX2B* mutations may have induced the initial injury, it is highly likely that the tissue alterations observed at the time of MRI assessment resulted not just from early mutations, but also from the damage inflicted by hypoxic and other sequelae from years of hypoventilation, inadequate perfusion, and associated physiological changes. Such a scenario offers the potential of intervention to reduce subsequent injury if the condition is detected sufficiently early. A portion of this discussion will examine the extent and sites of injury, the effects on autonomic nervous system and breathing control as a consequence of that injury, and the means by which the damage was inflicted, as well as potential interventions.

## Overview of injury

Prominent structural injury appeared in cortical and diencephalic sites, and midbrain, medullary and cerebellar regions in adolescent CCHS children (Kumar et al., 2005, 2008b, 2010, 2011; Macey et al., 2009, 2012a). These areas of injury overlap several classic autonomic regulatory sites, and include the hypothalamus and basal forebrain, basal ganglia, midline pons, locus coeruleus, raphe, nucleus of the solitary tract, ventrolateral medulla, and portions of the cerebellar cortex and deep nuclei. Among cortical regions, the insula, especially on the right side, the ventral medial frontal cortex, the anterior and posterior cingulate, and isolated areas within the parietal cortex show thinning or injury. The tissue changes are not confined to cortical areas and subcortical structures; substantial loss of fibers and changes in axonal integrity appear in the corpus callosum and in major axonal pathways between autonomic-related structures (Kumar et al., 2010, 2011). The structural deficits are accompanied by time-distorted or magnitude-impaired central functional responses to ventilatory and blood pressure challenges (Macey et al., 2005b; Ogren et al., 2010); those impaired brain responses are accompanied by distorted physiological response patterns.

**Figure 2:**
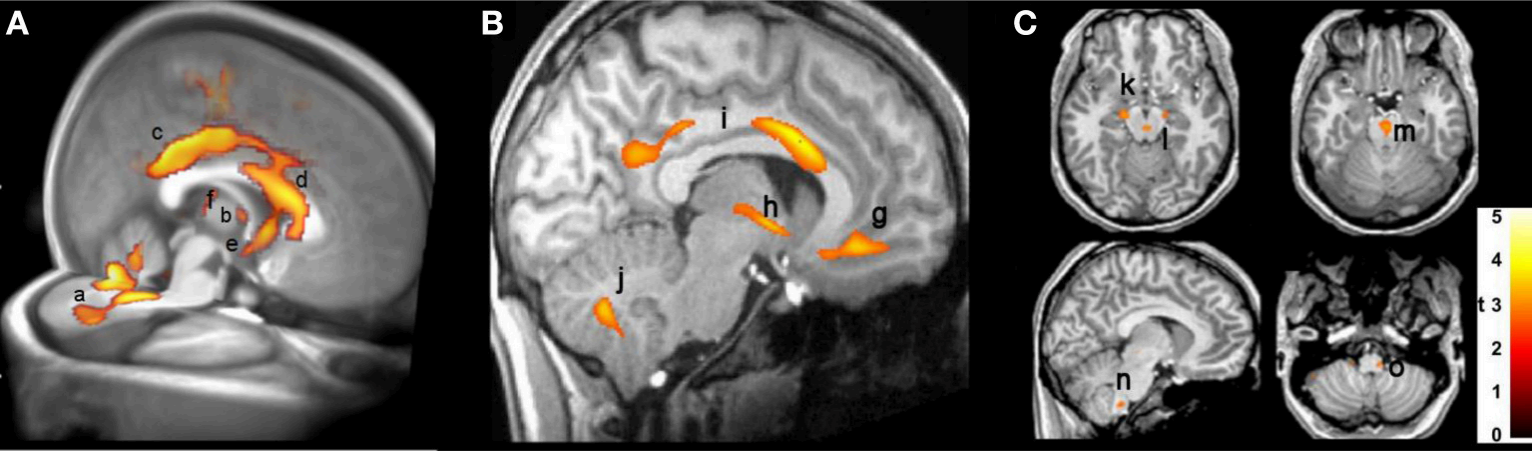
**(A)** 3D view of injury, based on diffusion tensor imaging, in cerebellum (a), anterior thalamus (b), posterior and anterior cingulate (c,d), hypothalamus and septum (e), and posterior thalamus (f) (Kumar et al., 2010). **(B)** Sagittal view, showing increased T2-relaxation values in (g) medial frontal cortex, (h) hypothalamic, (i) cingulate, and (j) cerebellar injury (Kumar et al., 2005); **(C)** axial diffusivity indications of injury in (k) cerebral peduncle and (l) periaqueductal gray, (m) midline raphe, and (n,o) ventrolateral medulla (Kumar et al., 2005). In this, and all following figures, images are displayed in neurological convention, i.e., left side of brain is left side of figure, and right brain is displayed on the right side.

The injury in CCHS patients in autonomic areas is so extensive as to be difficult to demonstrate in an overview image, but a representation of selected areas is shown in Figure 2, which reveals damage by T2 relaxation measures and diffusion tensor imaging procedures. Among the areas affected are fibers within the anterior and posterior cingulate, essential in cardiovascular control (Critchley et al., 2003; Shoemaker et al., 2015), the posterior thalamus, which plays a significant role in oxygen and breathing regulation (Koos et al., 1998, 2000, 2004), the anterior thalamus, part of the limbic network, the septum and hypothalamus (significant structures for mediating memory, rage, and a wide range of temperature, and hormonal regulation, respectively), and projections to the cerebellum, which exerts major dampening roles for extremes of blood pressure change (Lutherer et al., 1983; Chen et al., 1994). Midbrain and medullary areas, including the periaqueductal gray, dorsolateral pons, raphe, and locus coeruleus are also affected; these structures serve cardiovascular, respiratory, serotonin release, CO_2_ sensing, and arousal/attention roles (McGinty and Harper, 1976; Ni et al., 1990a,b; Nichols et al., 2008; Gargaglioni et al., 2010; Tomycz et al., 2010).

## Cortical damage and thinning

**Figure 3:**
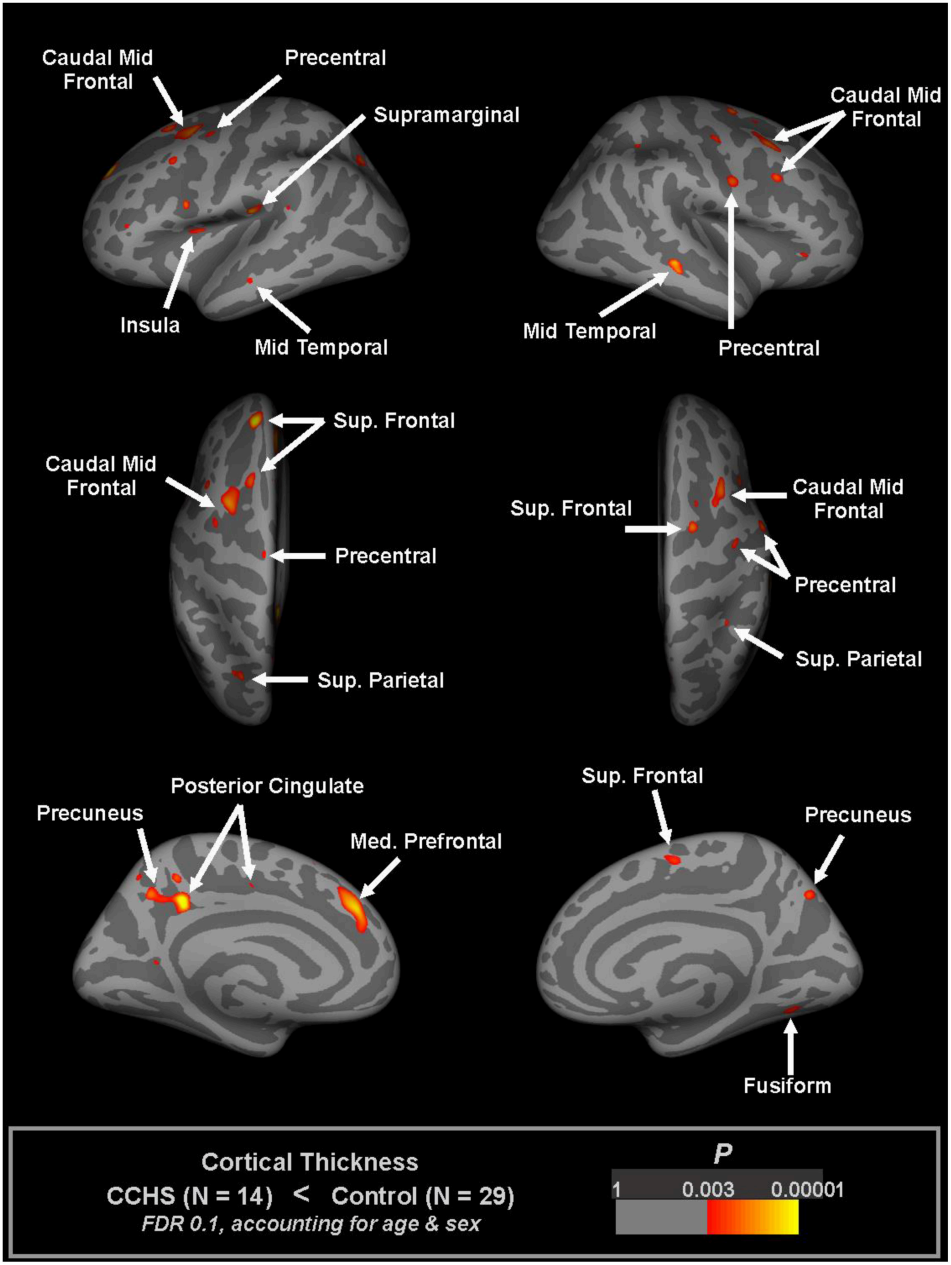
**Regions of significantly reduced cortical thickness in CCHS relative to control subjects, considering both age and sex (FDR 0.1), overlaid onto inflated pial surface (light gray gyral, dark gray sulcal)**. The principal thinning is found in insular, cingulate, and temporal cortices, and in motor strips (Macey et al., 2012a).

**Figure 4:**
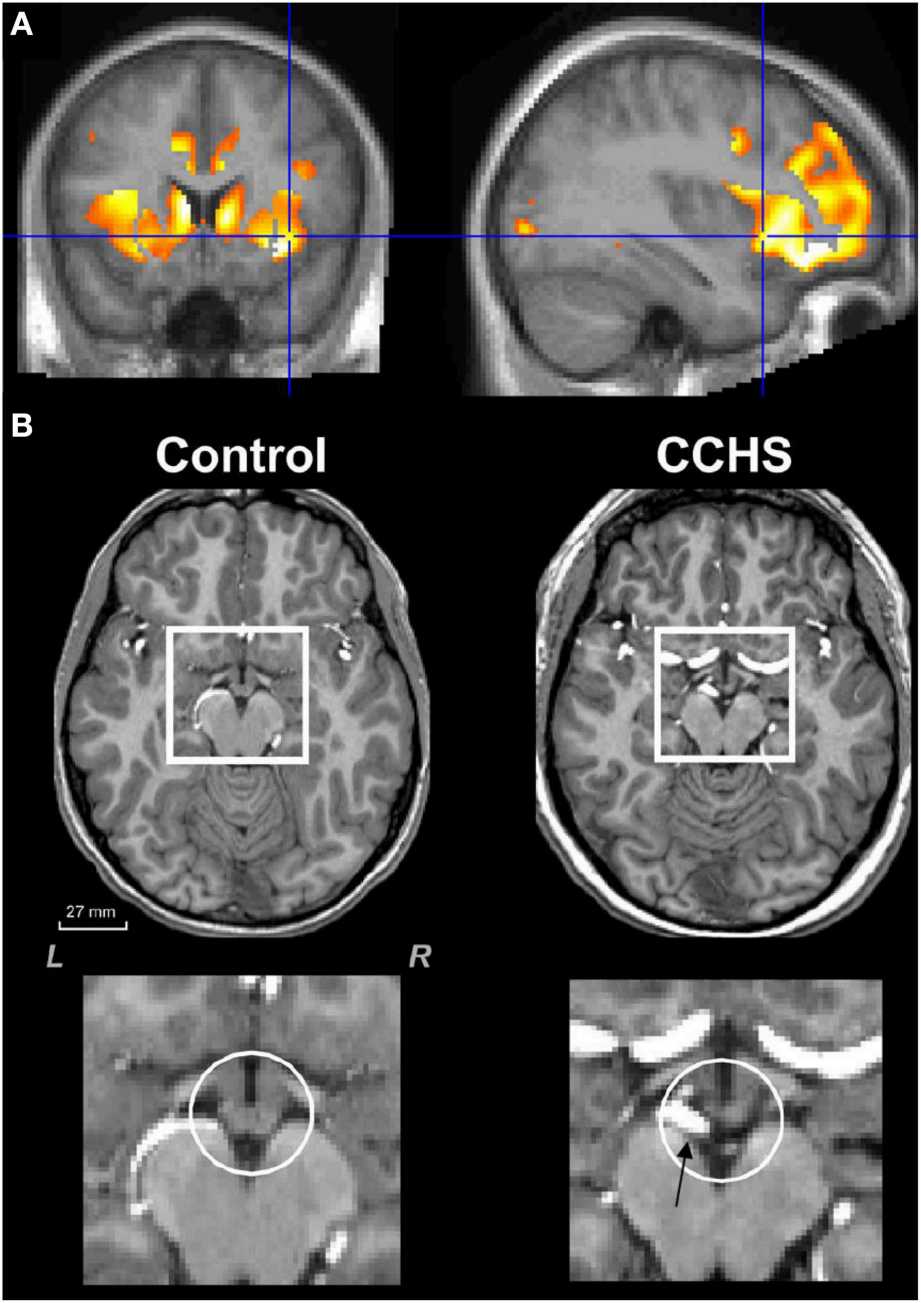
**(A)** Mean diffusivity measures of injury in CCHS indicating damage to the left and right insula, as well as to the cingulate cortex and to the caudate (Kumar et al., 2006). **(B)** Loss of mammillary body volume in a CCHS patient vs. a control; ventral arteries (arrow, lowest right panel) are also dilated in the condition. Group data are presented in Kumar et al. (2009a).

Thinning of the cerebral cortex appeared in many autonomic sites in CCHS, including the dorsomedial frontal cortex, anterior and posterior cingulate, medial prefrontal, insular, and anterior and lateral temporal lobes (Macey et al., 2012a; Figure 3). Other non-autonomic sites were also affected, including the motor strips and parietal cortex. Much of the cortical thinning in autonomic sites was expected from earlier-demonstrated fiber loss; the cingulum bundle, for example, is severely affected in CCHS (Kumar et al., 2005, 2010), and would certainly be associated with thinning in the anterior and posterior cingulate cortex. Similarly, the loss of integrity in the fornix from the hippocampus, as well as the hippocampal injury which alters other cortical projections, would contribute to the temporal cortex thinning. The insular cortex thinning was also expected, since earlier studies showed prominent injury (Figure 4).

The left and right insulae play different roles for autonomic regulation, depending on the nature of the autonomic challenge (Macey et al., 2012b), with the right side exerting more regulation over a cold pressor challenge. The anterior right insular cortex serves a prominent role in modulating the baroreflex (Kimmerly et al., 2005), and stroke injury to the left insula has been followed by enhanced sympathetic tone and myocardial infarction post-stroke (Oppenheimer et al., 1996; Cechetto and Hachinski, 1997; Laowattana et al., 2006). Injury or thinning of the insula may reduce its modulatory role on the hypothalamus and contribute to the enhanced sympathetic outflow. Both the left and right insulae are injured in CCHS (Kumar et al., 2012). The consequences of such injury are apparent at all ages, and especially in older children, where anxiety and depression are common. However, the most prominent characteristic rests with elevated and unresponsive sympathetic tone, as well as parasympathetic deficits, especially to parasympathetic outflow to the eyes, and a failure of vagal influences on heart rate variation (Woo et al., 1992). Injury to the insulae, as well as to the cerebellum and cingulate cortex, appears in obstructive sleep apnea (OSA) patients with depression and anxiety (Cross et al., 2008; Kumar et al., 2009b).

The right anterior cingulate is severely injured in CCHS, with the cingulum bundle, the principal body of fibers lying within the cingulate cortex undergoing major loss of integrity (Kumar et al., 2005). The cingulum bundle helps interconnect the amygdala and the anterior cingulate, with projections to the insula. The anatomical interactions between the amygdala, anterior cingulate, and insula have significant implications for the late-developing increased anxiety and depression, frequently found in CCHS. All three structures, as well as the fibers of the fornix, show alterations in other conditions characterized by high levels of anxiety and depression (Cross et al., 2008; Kumar et al., 2009b; Van Eijndhoven et al., 2009; Sprengelmeyer et al., 2011; Sacher et al., 2012).

Damage to the right anterior cingulate poses a more subtle autonomic regulation problem for CCHS. The right anterior cingulate plays a triggering role for urination (Blok et al., 1997). Children with CCHS have very poor fluid regulation, often drinking very large quantities of soda or water, but then requiring an order by a caretaker to urinate, at which time urination is excessive. The fluid regulation issue takes on additional importance with the high sympathetic tone in the condition, and the resultant profuse sweating, since that fluid loss carries with it water-soluble nutrients. Especially important in CCHS is loss of thiamine and magnesium, vital components for formation of ATP, essential for providing nutrients to cells (Singleton and Martin, 2001; Martin et al., 2003). Deficiencies in thiamine and magnesium are characteristic of conditions associated with a wide range of injuries to limbic and cerebellar structures, and especially the hippocampus and mammillary bodies (Kumar et al., 2008a, 2009c; Harper et al., 2014a). Reduced levels of thiamine are common in conditions of poor nutrition, impaired visceral absorption, high fluid loss from diuresis, profuse sweating, or diabetes (Thornalley, 2005; Harper, 2006). Those characteristics are common in chronic alcoholism (patients are prone to visceral malabsorption, poor nutrition, due to few calories, except for those from alcohol), Beriberi (long-term malnutrition from thiamine and magnesium deprivation), HF (diuresis, malabsorption, and high sympathetic tone leading to excessive sweating), OSA (high sympathetic tone, increased sweating, high incidence of diabetes) (World Health Organization and United Nations High Commissioner for Refugees, 1999; Martin et al., 2003; Thornalley, 2005; Harper, 2006; Harper et al., 2014a). The factors leading to vital nutrient depletion in CCHS are shared with a number of other conditions, and the interventions for protection in those conditions are well-described; recognition of nutrient depletion in CCHS, however, is seldom recognized.

## Hippocampus, fornix, and mammillary bodies

**Figure 5:**
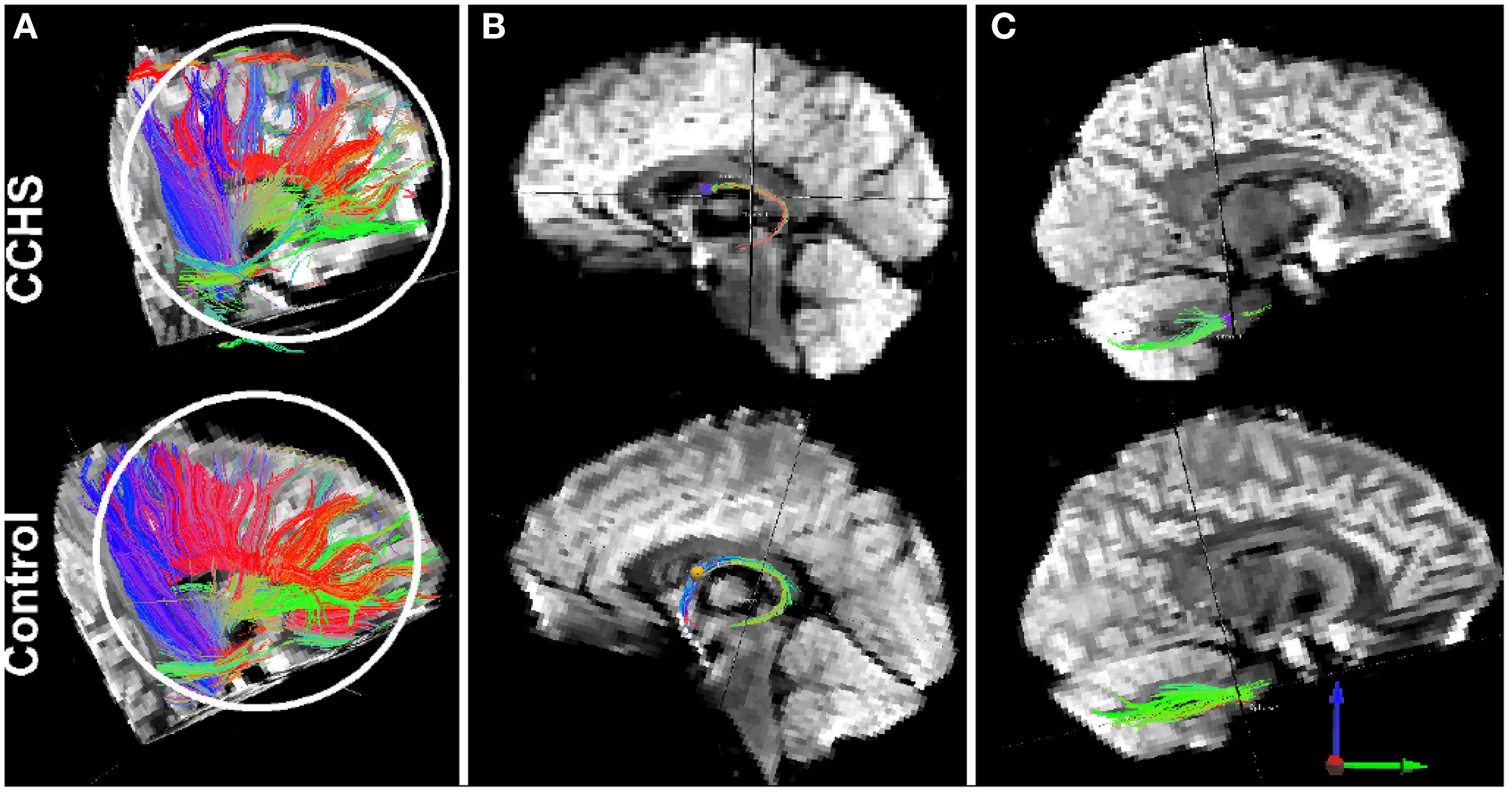
**Loss of fibers, as determined by diffusion tensor imaging tractography procedures, in single CCHS and Control subjects**. **Panel (A)** shows overall fiber loss between multiple sites; **Panel (B)** shows the loss of anterior projecting fibers of the fornix (loss of blue fibers), and **panel (C)** shows loss of fibers from comparable projection sites in the pons to the cerebellum. T1-weighted data confirming group fiber loss are outlined in Kumar et al. (2009a).

The hippocampal injury found in CCHS patients may lead to autonomic impairments, perhaps from the role of this structure as part of a ventral medial frontal cortex-hippocampal blood pressure regulatory circuit (Shoemaker et al., 2015). The structure earlier has shown a role in modulation of blood pressure, as demonstrated in both humans (Harper et al., 2000) and animals (Ruit and Neafsey, 1990). A major output path of the hippocampus, the fibers of the fornix, show substantial loss of integrity in CCHS (Figure 5), and a target of those fibers, the mammillary bodies, is significantly diminished in volume in CCHS (Figure 4; Kumar et al., 2009a). Additionally, the anterior thalamus, which receives projections from the mammillary bodies, is also affected in the syndrome (Figure 2). The injury in CCHS is shared with other sleep-disordered breathing conditions, including OSA and HF, and other syndromes, such as Wernicke-Korsakoff's syndrome stemming from chronic alcoholism (Nahum et al., 2015), and Beriberi (Hahn et al., 1998), all conditions in which intestinal malabsorption, severe water loss, and poor thiamine and magnesium intake are characteristic.

## Raphe, locus coeruleus injury

The raphe system is significantly affected in CCHS, as shown in Figure 2. The consequences of such injury are severe for autonomic regulation. The raphe system is the source of serotonergic neurons, which influence the cutaneous vascular bed, thus compromising temperature control (Nalivaiko and Blessing, 2001; McAllen and Schwartz, 2010), as well as pain regulatory sites in the spinal cord. The medullary raphe also is a relay for stress-induced cardiac sympathetic activity (Zaretsky et al., 2003), and sends projections to more-rostral areas influencing cognitive and affective behaviors (O'Hearn and Molliver, 1984). The raphe is also a source of thyrotropin-releasing hormone (TRH) neurons (Barnes et al., 2010; in addition to the hypothalamus), and thus can modulate a chain of reactions affecting glucose control. The effects of distortion in 5-HT neurons found on the nearby vasculature (Figure 4), can be seen in the extreme resting dilation of ventral arterial vessels in the condition, an aspect that likely contributes to impaired chemoreception (Bradley et al., 2002). Undoubtedly, the altered serotonergic influences resulting from impaired raphe cells will be reflected in depression and anxiety symptoms, which are heavily influenced by serotonergic action.

*Phox2b* is heavily expressed in the locus coeruleus of a murine model, as shown in Figure 1. If humans share similar expression, the consequences to CCHS are severe, as noted in one CCHS patient who succumbed, showing substantial histopathology in this nucleus (Tomycz et al., 2010). The injury has been repeatedly demonstrated as well in structural MR images (Kumar et al., 2005, 2012; Macey et al., 2009), and in fMRI responses to hypercapnia (**Figure 7**). The consequences of losing noradrenergic innervation from locus coeruleus injury are significant, since, in addition to CO_2_ sensing, roles for focusing, attention, and general arousal are affected (Sara and Bouret, 2012).

## Axonal injury

**Figure 6:**
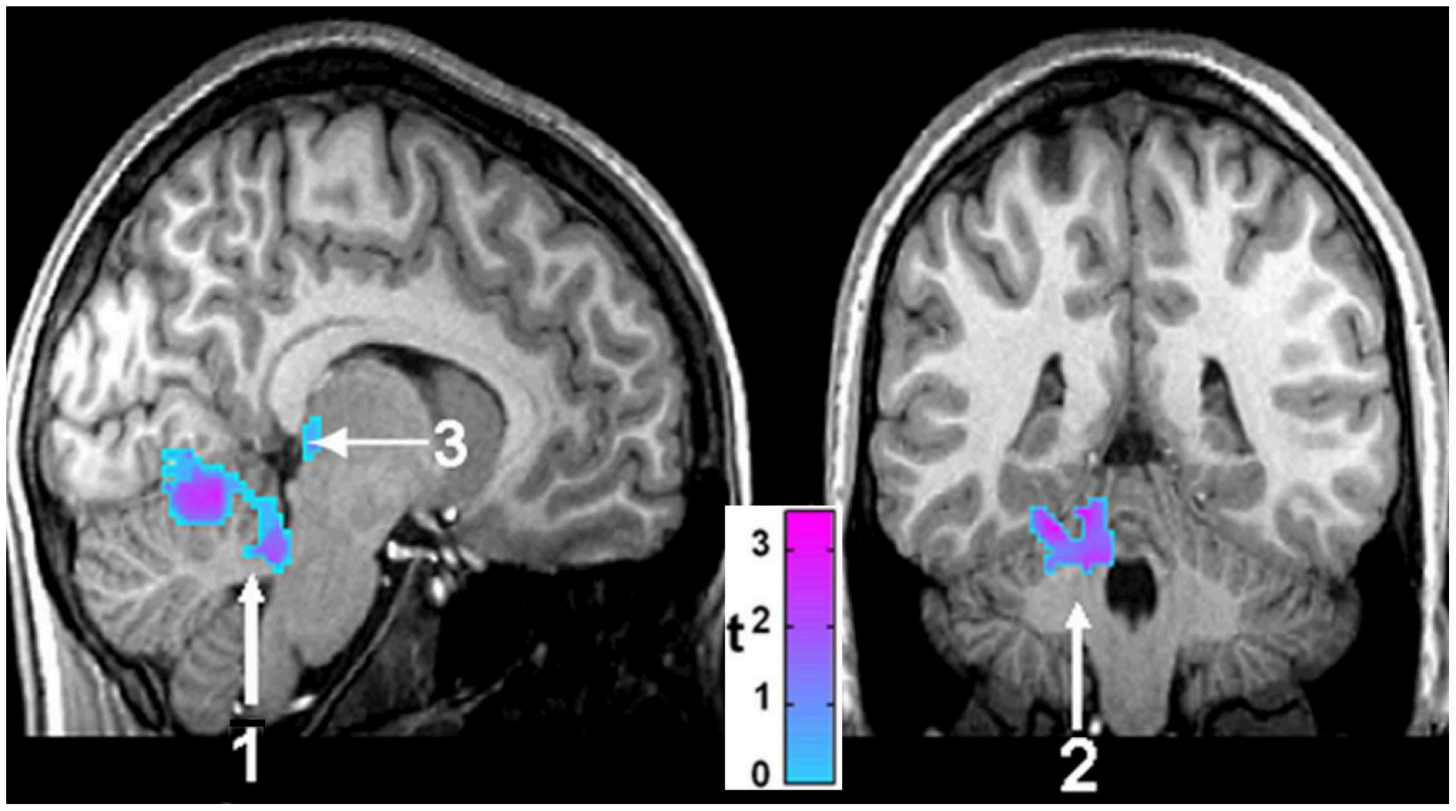
**Sagittal (left) and coronal (right) views of brain areas of 14 CCHS children which show significant differences from 14 controls in response to mild hypoxia (15% O_2_, 85% N_2_)**. The areas include the cerebellum (1, 2) and the posterior thalamus (3) (Macey et al., 2005b).

The injury in CCHS is not confined to nuclear structures. Even more prominent is the extent of axonal injury, which appears across the entire brain, and is especially prominent in the corpus callosum (Kumar et al., 2010, 2011), fibers between limbic areas (Kumar et al., 2010), and projections from the pons to the cerebellum (Figure 6). A remarkable aspect is the asymmetry of the fiber injury, just as portions of the nuclear damage are lateralized. The lateralization of injury is particularly apparent in pontine-cerebellar projections, a concern of importance to the autonomic community because of the substantial role that the cerebellum plays in dampening extremes of blood pressure (Lutherer et al., 1983) and time-coordinating somatic responses to blood pressure changes (Ogren et al., 2010). Figure 6C illustrates the loss of integrity of pontine projections to the cerebellum, with significantly more fiber loss on the left side over the right, relative to a healthy control. Other prominent axonal loss is found in the cingulum bundle, fibers of the fornix, and projections to the frontal cortex from other sites (Kumar et al., 2005, 2010). The axonal loss across the corpus callosum is not uniform, with more fiber loss in anterior and posterior regions (Kumar et al., 2011); the latter is significant for integration of autonomic regulation of the pupils with eye movements, which are affected in CCHS. Somatic motor pathways such as the descending motor pathways of the pyramidal tract, were largely spared (Kumar et al., 2008b).

## Glucose dyscontrol from altered autonomic regulation

The significant injury to hypothalamic structures, as well as to cortical regions influencing hypothalamic output, and the substantial injury to the raphe system with its TRH neurons (Kumar et al., 2008b), together with injury to parasympathetic vagal influences on the viscera and the high, unvarying sympathetic tone, suggest a potential for impaired glucose regulation. The concern in CCHS is hypoglycemia (Farina et al., 2012), with hypoglycemic seizures in the syndrome occasionally mistaken for hypoxic seizures. The high sympathetic tone will alter normal regulation of glucagon levels, and the role of the sympathetic system in modulating insulin release also will be impacted. TRH release from the damaged raphe will likely be diminished, affecting the primary role that the hormone exerts on insulin release, with potential hyperglycemic effects (Yang et al., 2002). The contrasting influences on hyper- and hypo-glycemia lead to complex interactions influencing glucose control. The evidence of impaired glucose regulation should not be ignored when considering the presence or progression of neural tissue injury in another sleep-disordered breathing condition, OSA. The presence of Type 2 diabetes substantially increases the extent of neural injury in patients with OSA (Harper et al., 2012). Although CCHS children more frequently show hypoglycemia rather than elevated glucose levels characteristic of Type 2 diabetes, epochs of hypoglycemia are also damaging to neural tissue.

## Central functional impairments to autonomic challenges

The structural injury in autonomic sites in CCHS affects both the extent and timing of central responses to autonomic challenges. The consequences are frequently found in normal activities that trigger a change in blood pressure, such as straining to physical effort, which is often accompanied by syncope in the condition, suggestive of a delayed or inadequate ability to maintain cerebral perfusion. The role of timing and extent of neural responses to blood pressure manipulation is best evaluated with a carefully timed autonomic challenge that recruits both sympathetic and parasympathetic responses, such as the Valsalva maneuver. Valsalva maneuvers in CCHS demonstrate an often-lateralized diminution or phase-shifting of responses in multiple autonomic sites throughout the brain, from limbic sites, such as the amygdala and hippocampus, to cerebellar areas (**Figure 8**). The responses are typically substantially diminished (e.g., all structures in **Figure 8**), inverted (e.g., amygdala), or time-distorted (e.g., right amygdala, left insula recovery, right ventral cerebellum recovery). More insidious for autonomic control was the asymmetry of impaired central responses in particular structures; the unilateral diminution of responses was particularly marked in the left amygdala and hippocampus **Figures 8A,B**, and in the right ventral cerebellum **Figure 8D**. Since the magnitude of responses has the potential to modify the extent of sympathetic outflow, with the cerebellum exerting dampening roles on such outflow, the lateralized impaired central responses in CCHS provide ideal circumstances for development of dangerous cardiac arrhythmia, such as long Q-T syndrome (Schwartz et al., 1976). Prolonged sinus intervals are typical in more-severe cases of CCHS, with sudden death common in the condition (Gronli et al., 2008). Pacing interventions to prevent extreme bradycardia are standard practice for severely-affected individuals.

## Posterior thalamus and hypoxia recovery

**Figure 7:**
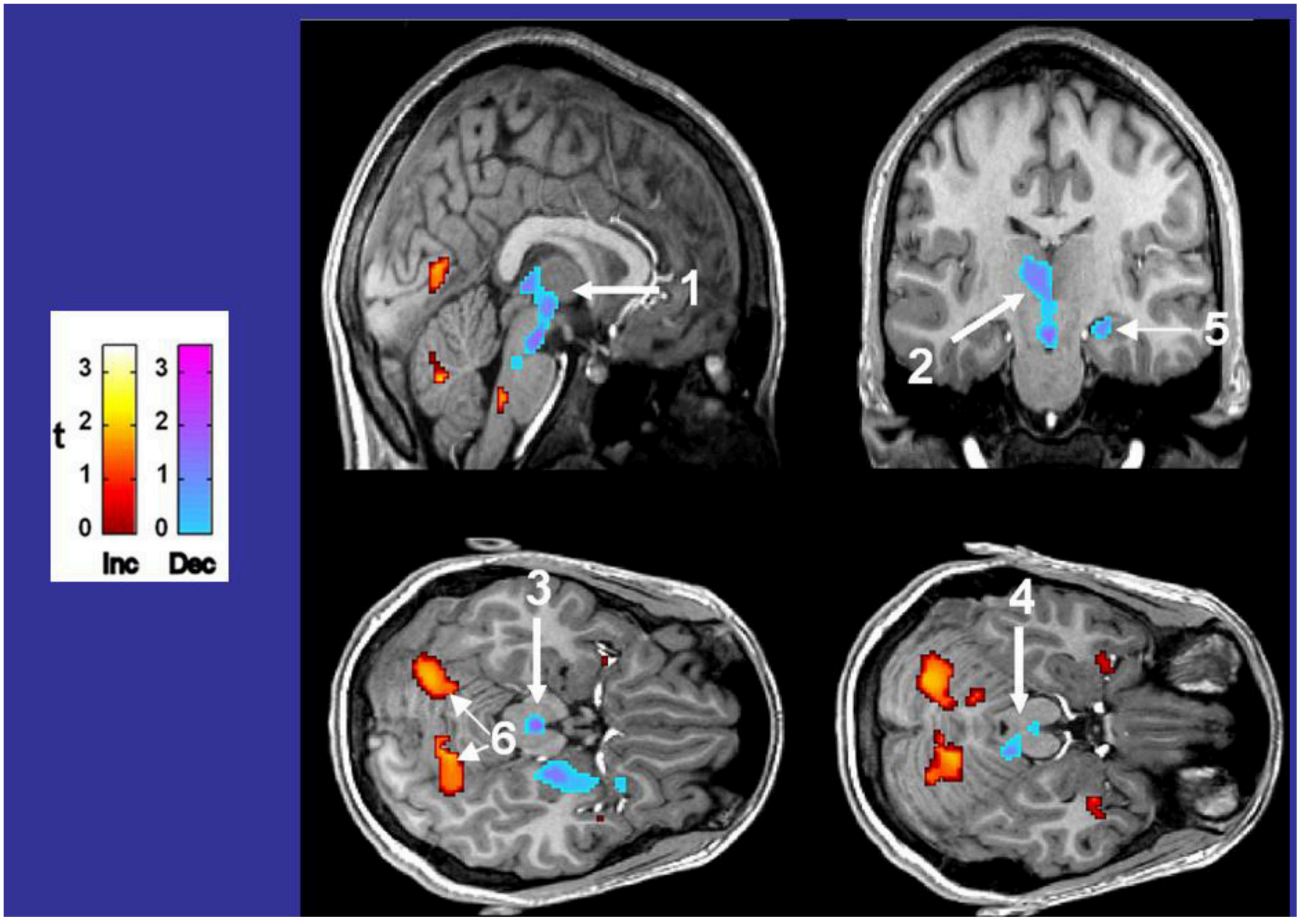
**Differences in fMRI responses to 5% CO_2_/95% O_2_ in 14 CCHS subjects vs. age- and gender-matched 14 controls, with warm colors showing increased (Inc) values, and cool colors showing decreased (Dec) values in CCHS from controls**. The responsive areas include the posterior thalamus, extending to the ventral midbrain (1, 2), the periaqueductal gray (3), the dorsolateral pons, including the locus coeruleus (4), the hippocampus (5), and the cerebellar cortex (6). Derived from Harper et al. (2005b).

A remarkable aspect of CCHS is the absence of the perception of breathlessness, i.e., dyspnea, in addition to deficient ventilatory responses to both hypercarbia and hypoxia. The loss of the sense of breathlessness to high CO_2_ presumably stems from insular, cingulate and cerebellar injury (Banzett et al., 2000; Peiffer et al., 2001), while the failure to cope with low oxygen likely stems from the structural damage in the posterior thalamus (Figure 4), a finding supported by deficient fMRI responses to hypoxic challenges in CCHS (Macey et al., 2005b; Figure 7). The insensitivity to hypoxia appears even during waking, when caretakers must often forcefully direct their charges to breathe when they are sitting quietly. Substantial evidence links the posterior thalamus to oxygen regulation roles, as shown by lesion and stimulation studies in the fetal lamb (Koos et al., 1998, 2004). The posterior thalamic injury is especially a concern, given the findings that gray matter volume loss appears in that area in people with epilepsy who have succumbed, or are at risk for sudden death in epilepsy (SUDEP; Wandschneider et al., 2015). Sudden death in CCHS frequently occurs in conjunction with absence of appropriate ventilatory support.

**Figure 8:**
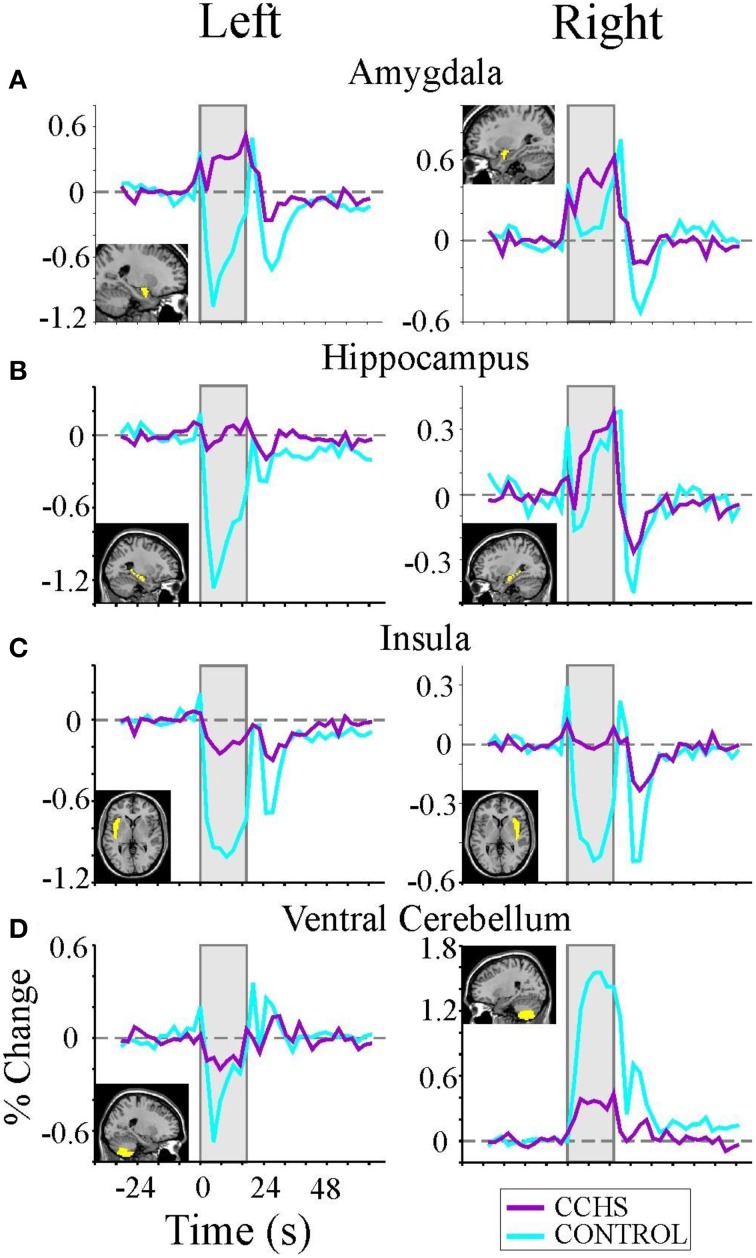
**(A–D)** Left and right rostral/limbic brain and ventral cerebellum fMRI responses to four averaged Valsalva maneuvers (shaded areas) in nine CCHS subjects and 25 controls, illustrating the significant phase shifts and reversals, and muting of signals in CCHS cases, and the lateralized nature of changes in particular structures. Mean signal time trends are averaged over the four Valsalva challenge periods, 17.5 s each. Signals are in percent change, relative to baseline. Inset panels show the voxels of interest corresponding to each mean signal time trend (Ogren et al., 2010).

The hallmark of the CCHS condition is ventilatory insensitivity to CO_2_. Exposure to 5% CO_2_ elicits pronounced fMRI signal changes in a number of brain areas (signals corrected for global changes induced by CO_2_), including the posterior thalamus, ventral midbrain, and cerebellum (Harper et al., 2005b). In addition, the dorsal lateral pons, periaqueductal gray, and hippocampus show pronounced signal differences from controls (Figure 8). The findings demonstrate the role of non-traditional structures, e.g., cerebellum, in mediating responses to hypercapnia, emphasize the contribution of the posterior thalamus in mediating both responses to CO_2_ and hypoxia, and add to the concern that injury to the posterior thalamus may contribute to the high incidence of sudden death in CCHS in a fashion similar to that described for SUDEP earlier (Wandschneider et al., 2015).

## Implications of lateralized injury on autonomic regulation

A large proportion of the injury found in CCHS is lateralized, with preferential loss of tissue or alterations in water diffusion, or greater loss of fiber integrity on one side of the brain over the other. Such lateralization includes the preferentially right-sided insular injury (although both sides are affected), the right-sided cingulate and ventral medial frontal cortex damage, the left-sided mammillary body volume loss, the left-sided ventral medullary injury, and the left-sided cerebellar fiber damage (Kumar et al., 2009a, 2010; Harper et al., 2014b). Even if functional roles for the right and left sides of the brain were equivalent, the asymmetry of injury would be of significant concern. For example, in the case of the ventrolateral medulla, unilateral injury raises the potential for influences on medullary sympathetic outflow to be enhanced to one side of the intermediolateral column of the spinal cord over the other, thereby increasing the possibility of asymmetric influences on the cardiac ganglia, and greatly increasing the possibility of arrhythmia (Schwartz, 1998). The lateralization of injury is also superimposed on asymmetry of functional autonomic roles for structures. The right insular cortex serves a much greater role in sympathetic regulation, with the left regulating parasympathetic action to a larger degree (Oppenheimer, 2006). It is the right ventral medial frontal cortex that precedes activation of the left hippocampus in response to a blood pressure challenge (Shoemaker et al., 2015), and it is the right anterior cingulate that is involved in initiating urination (Blok et al., 1997). Other, yet undescribed autonomic functional lateralization of actions may be present, but it is apparent that, like some other somatic or cognitive functions, such as language, which is principally represented on the left side (Ojemann et al., 1989), control of autonomic functions is often lateralized, with the implication that asymmetric appearance of injury can have much more profound outcomes if reorganization of function is unavailable for that side of the brain.

## Neuroprotection in CCHS

The brain injury found in CCHS appears to progress, at least in a small sample that was followed over several years, and in selected areas (Kumar et al., 2012). The mechanisms underlying that progression are unknown. It is unclear whether the changes emerge from some unknown, continued action of the *PHOX2B* mutations, from continuing exposure to hypoxic episodes, or from uncontrolled variation in blood pressure that accompanies the syndrome and would be especially manifested in apnea periods, or by some other process. The progression in injury was especially prominent in areas affecting mood and autonomic function, and included the insular, frontal and cingulate cortices, cerebellum, basal ganglia, and dorsal medullary regions (Kumar et al., 2012). The progression of injury is not precisely mirrored by impaired cognitive or mood function; individual CCHS cases have shown high performance in cognitive and executive function in adult years; however, some high-functioning cases have succumbed to consequences, e.g., suicide, of later-developing mood disorders, or to late-onset cardiac arrhythmia. Analogous, if later developing, patterns are found in OSA and HF, both of which show development of anxiety and depression in affected patients; these emotional conditions are suspected of arising from cingulate and insular damage (Kumar et al., 2005; Macey et al., 2012a). A major contribution to the later-onset neural injury in CCHS, as well as OSA and HF, could arise from ancillary characteristics of the primary syndromes that must be controlled to prevent onset of very serious, potentially fatal consequences.

The high sympathetic outflow in CCHS appears to lead to several of the serious late-developing sequelae in CCHS. The high sympathetic drive is found in other conditions with sleep disordered breathing, including OSA and HF (Fatouleh et al., 2014; Florea and Cohn, 2014). The sustained high, and often asymmetric sympathetic tone provides a significant risk for cardiac arrhythmia and impaired perfusion to critical brain areas, including autonomic sites. However, the enhanced sweating from high sympathetic tone, combined with poor fluid regulation, can deplete water-soluble nutrients essential for neuroprotection. The consequences have been described earlier for other conditions, with brain structural changes characteristic of loss of thiamine and magnesium (Kumar et al., 2008a, 2009c), and are similar to those found in malnutrition, Beriberi, and chronic alcoholism (World Health Organization and United Nations High Commissioner for Refugees, 1999). The neural injury includes greatly reduced mammillary body volumes, hippocampal, and cerebellar damage, among other brain structural changes (Harper, 2006; Pearce, 2008). Thiamine is essential for glucose processing into cellular energy through ATP, and depends on magnesium for that conversion (Martin et al., 2003). Although low magnesium and thiamine levels have not been confirmed for CCHS, they have for OSA (Harper et al., 2014a); such depletion places cardiac patients at high risk for sudden death (Booth et al., 2003; Peacock et al., 2010). CCHS patients show the hallmarks of similar essential nutrient depletion with reduced mammillary body size (Kumar et al., 2009a), as well as hippocampal and cerebellar injury (Kumar et al., 2005; Macey et al., 2009). Thiamine and magnesium supplements may prove to be exceptionally low cost interventions to reduce further injury to brain structures, especially to autonomic structures, such as the hippocampus, cerebellar Purkinje cell projections to the deep “autonomic” nuclei, the fastigial nuclei, and the insular cortices.

Development of anxiety and depression is an increasing concern in CCHS as children mature, an outcome likely resulting from injury to “autonomic” regions of the insular cortices, cerebellum, and anterior cingulate; those autonomic areas share roles in mediating depression, with the anterior cingulate showing an especially prominent role in that mood disorder (Sacher et al., 2012). Development of mood and anxiety issues typically requires some time in affected children, and possibly could result from progressive injury in CCHS (Kumar et al., 2012). Aggressive attention to neuroprotection may be beneficial in slowing such mood change development.

## Perspectives and summary

CCHS provides an exceptional “Experiment of Nature” to reveal the role of particular brain structures in mediating autonomic activity, as well as the means by which these structures interact with breathing and other physiological and behavioral functions. The initial target of mutations in *PHOX2B* are structures and processes essential for chemoreception and vascular regulation, and the condition has provided insights into brain structures typically not identified as serving respiratory or autonomic functions. Thus, roles for the posterior thalamus, cerebellum, and portions of the hippocampus in processing ventilatory responses to low oxygen or hypercarbia are not usually recognized in the cardiorespiratory community, but the CCHS evidence demonstrates the significant contributions of these structures. Similarly, the role of the sense of breathlessness in providing a breathing drive is usually not recognized, but the absence of that function is especially apparent in affected children, and likely stems from damage to insular, cingulate, and cerebellar structures that accompanies the syndrome. An overriding concern, however, is the compromised sympathetic nervous system activity, induced by structural changes, which has the potential to affect a vast array of hormonal and metabolic actions, interfering with a wide range of glucose regulation and neurotransmitter functions through indirect consequences to vascular and neuroregulatory influences.

Since the initial injury to sympathetic regulatory and other systems can lead to progressive damage, interventions to slow, or halt the progression of damage and subsequent behavioral deterioration are mandatory. One simple and low cost intervention is to ensure that neural processes are not starved, a failing arising from the extreme sympathetic excitation/nutrition loss processes. That potential therapy is only one example of possible mechanisms to forestall later-developing mood and cardiovascular consequences resulting from injured neural structures.

Current interventions for providing breathing or autonomic support in CCHS are crude. Ventilatory assistance consists principally of poorly-regulated positive pressure ventilation or phrenic pacemakers, which are relatively unresponsive to momentary demands for gas exchange during sleep. The only autonomic support rests with cardiac pacemakers to prevent excessively long cardiac pauses. However, several interventions for CCHS are on the horizon, and make use of sensory and motor processes which are preserved, and which can substitute activation of existing reflexes to replace lost functions. The descending voluntary motor pathways are relatively preserved in CCHS (Kumar et al., 2008b), and proprioceptive sensory systems also are functional. Those intact processes can be recruited to overcome, or neutralize some of the deficits found in chemoreception and failed autonomic systems. The most obvious of these interventions is the use of proprioceptive action from limb movement to restore breathing (Gozal and Simakajornboon, 2000), and inherent reflexes which couple movement with breathing (Harper et al., 2005a; Macey et al., 2005a). Several other approaches can be implemented to correct autonomic failings, with the development of neuromodulation procedures to alter vagal and other autonomic nerve activity. Several aspects of cardiovascular control must be corrected in CCHS, including modulation of heart rate and blood pressure by breathing. Such interventions are currently non-existent, but the coupling is essential for appropriate perfusion of the brain and prevention of arrhythmia. The future appears promising for the development of new interventions.

### Conflict of interest statement

The authors declare that the research was conducted in the absence of any commercial or financial relationships that could be construed as a potential conflict of interest.

## Footnotes

^1^GENSAT The Gene Expression Nervous System Atlas (GENSAT) Project. New York, NY: The Rockefeller University. Available online at: http://www.gensat.org/imagenavigatorfileselection.jsp?gene.id=1731&allages=true (Accessed June 30, 2015).
